# Baseline Cerebro-Cerebellar Functional Connectivity in Afferent and Efferent Pathways Reveal Dissociable Improvements in Visuomotor Learning

**DOI:** 10.3389/fnins.2022.904564

**Published:** 2022-05-26

**Authors:** Yi-Cheng Lin, Yun R. Lien, Shang-Hua N. Lin, Yi-Chia Kung, Chu-Chung Huang, Ching-Po Lin, Li-Hung Chang

**Affiliations:** ^1^Institute of Neuroscience, National Yang Ming Chiao Tung University, Taipei, Taiwan; ^2^Taipei Municipal Gan-Dau Hospital, Taipei, Taiwan; ^3^Institute of Brain and Education Innovation, School of Psychology and Cognitive Science, East China Normal University, Shanghai, China; ^4^Shanghai Center for Brain Science and Brain-Inspired Technology, Shanghai, China; ^5^Institute of Philosophy of Mind and Cognition, National Yang Ming Chiao Tung University, Taipei, Taiwan

**Keywords:** rTMS, cerebellum, visuomotor coordination, functional connectivity, dentate nucleus, dentato-thalamo-cortical pathways, cerebro-cerebellar connections

## Abstract

Visuomotor coordination is a complex process involving several brain regions, primarily the cerebellum and motor cortex. Studies have shown inconsistent resting-state functional magnetic resonance imaging (rsfMRI) results in the cerebellar cortex and dentate nucleus of the cerebro-cerebellar connections. Echoing anatomical pathways, these two different cerebellar regions are differentially responsible for afferent and efferent cerebro-cerebellar functional connections. The aim of this study was to measure the baseline resting-state functional connectivity of different cerebellar afferent and efferent pathways and to investigate their relationship to visuomotor learning abilities. We used different cerebellar repetitive transcranial magnetic stimulation (rTMS) frequencies before a pursuit rotor task to influence visuomotor performance. Thirty-eight right-handed participants were included and randomly assigned to three different rTMS frequency groups (1 Hz, 10 Hz and sham) and underwent baseline rsfMRI and pursuit rotor task assessments. We report that greater baseline functional connectivity in the afferent cerebro-cerebellar pathways was associated with greater accuracy improvements. Interestingly, lower baseline functional connectivity in the efferent dentato-thalamo-cortical pathways was associated with greater stability in visuomotor performance, possibly associated with the inhibitory role of the dentate nucleus and caused a reduction in the efferent functional connectivity. The functional dissociation of the cerebellar cortex and dentate nucleus and their connections, suggests that distinct mechanisms in the cerebellum regarding visuomotor learning, which should be investigated in future research.

## Introduction

The cerebellum plays a critical role in visuomotor coordination and procedural learning processes (Manto et al., 2012; Raymond and Medina, 2018). The cerebellum detects a mismatch between motor commands sent from the cerebral cortex and sensory feedback generated by movement through afferent connections and then returns the error signal through efferent connections that modulate subsequent motor commands (Streng et al., 2018). It has been well accepted that the cerebellum and its cerebro-cerebellar input and output pathways contribute to visuomotor learning (Izawa and Shadmehr, 2011). The cerebro-cerebellar functional connectivity was involved in visuomotor learning in humans. Some studies have shown that the cerebro-cerebellar network was positively correlated with cerebellum-dependent learning tasks (Miall et al., 2001; Muller et al., 2002; Della-Maggiore et al., 2017; Shao et al., 2019). However, other studies have shown a negative correlation between changes in functional connectivity in the cerebellar cortex and M1 after motor learning (Vahdat et al., 2011; Mawase et al., 2017). Both the cerebellar cortex and the deep cerebellar nuclei (DCN) (which form the main efferent pathway from the cerebellum to the cerebral cortex, for example, the dentate nucleus) are involved and regulate visuomotor coordination. Furthermore, dynamic changes in the cerebellar cortex increased in early learning and decreased in late learning stages and were correlated with learning stability (decreased tracking errors) (Floyer-Lea and Matthews, 2004). An opposite pattern of dynamic changes was noted in the dentate nucleus, which showed low activation in the early stage, increased with performance improvements, and then remained relatively stable (Floyer-Lea and Matthews, 2004). These studies of resting-state cerebro-cerebellar functional connectivity studies showed inconsistent results (positively and negatively correlated with the cerebellum-dependent learning task), which indicated differences in the cerebellar cortex and DCN. In addition, echoing the anatomical cerebellar pathways, it is well known that these two different cerebellar regions are responsible for different directions of cerebro-cerebellar connections. The cerebral cortex to cerebellar cortex connections are mainly afferent cerebro-ponto-cerebellar pathways, and the cerebral cortex to DCN connections are mainly efferent dentato-thalamo-cortical pathways. Whether afferent and efferent connections work together or are responsible for different domains in the visuomotor learning process remains to be elucidated.

As the cerebellar afferent and efferent pathways serve different functions, their functional connectivity may differ on cerebellar coordination abilities. By dividing functional connectivity into efferent and afferent pathways, we might demonstrate the effects of functional connectivity on cerebellar coordination abilities. Therefore, we measured the cerebro-cerebellar connections for the afferent cerebro-ponto-cerebellar pathways and the dentato-cortical connections for the efferent dentato-thalamo-cortical pathways by setting regions of interest (ROIs) in the cerebellum cortex and the DCN, especially the dentate nucleus. Motor learning can modulate subsequent activity within resting networks (Logothetis et al., 2001; Albert et al., 2009), and resting-state activity supports introspective thought, processes previous experiences, and supports responses to future events (Miall and Robertson, 2006; Raichle and Snyder, 2007). By testing baseline cerebro-cerebellar functional connectivity (Mawase et al., 2017), we might predict visuomotor learning abilities. We aimed to measure the baseline levels of functional connectivity in cerebellar pathways and their relationships to visuomotor learning abilities.

In our study, a pursuit rotor task was applied to assess visuomotor learning abilities (Lien et al., 2022). We calculated improvements in performance at the minimum distance from the target point as indices of accuracy and the trial-to-trial variability of distance from the target as indices of stability (Lien et al., 2022). Visuomotor learning performance can be modulated by repetitive cerebellar transcranial magnetic stimulation (rTMS) (Koch, 2010; Lee et al., 2014; Tang et al., 2016; Rastogi et al., 2017). Therefore, we used different frequencies (1 Hz and 10 Hz) of cerebellar rTMS to influence visuomotor coordination abilities in the pursuit rotor task. We assumed that the baseline cortico cerebellar functional connectivity, especially the connections corresponding to the afferent and efferent circuits, are able to predict visuomotor learning abilities but may differentially contribute to performance. In addition, cerebrocerebellar circuit neuromodulation, such as cerebellar rTMS, may be effective for the control of motor symptoms in patients with cerebellar lesions or visuomotor deficits (Miterko et al., 2019). The neuromodulation of the cerebellum based on this knowledge with potential therapeutic applications for different cerebellar disorders.

## Materials and Methods

### Participants

All right-handed participants were randomly assigned to three groups and thirty-eight participants were included for analysis: the cerebellar rTMS – 1 Hz group (*n* = 12; age = 22.2 ± 1.3 years; 5 males, 7 females), 10 Hz group (n = 15; age = 22.8 ± 1.5 years; 6 males, 9 females), and sham group (*n* = 11; age = 23.4 ± 2.6 years; 6 males, 5 females; see Supplementary Table 1). In addition, eleven participants (five in sham, four in 1 Hz group, and two in 10 Hz group) were excluded because of poor image quality and ten participants (six in sham, two in 1 Hz group, and two in 10 Hz group) were excluded because they did not meet the minimum requirement of accuracy in the visuomotor task. No significant differences were found between the three groups in age, sex ratio, or educational year. All participants were assessed by clinical neurologist, and those who had abnormal neurological issues were excluded. This study was approved by the institutional review board of National Yang Ming Chiao Tung University. All participants gave their informed consent and underwent all experiments at National Yang Ming Chiao Tung University.

### Experimental Procedure

All participants underwent a baseline magnetic resonance imaging (MRI) session that acquired T1-weighted images and resting-state functional MRI (rsfMRI) to determine the regions that deliver stimulation and to calculate functional connectivity, respectively. Before the behavioral and rTMS sessions, the participants underwent the phosphene threshold (PT) measurements in order to determine the reference intensity for cerebellar rTMS in the rTMS session. The experimental procedure is shown in Supplementary Figure 1. During the behavioral and rTMS sessions, the participants completed behavioral tasks (15∼20 min per session) before and after the cerebellar rTMS interventions (less than 10 min per session).

### Magnetic Resonance Imaging Data Acquisition

All imaging data acquisition was performed at the National Yang Ming Chiao Tung University using a 3T Siemens Magnetom Tim Trio MRI scanner (Erlangen, Germany) with a 32-channel head coil. First, high-resolution T1-weighted MRI images were acquired with a three-dimensional magnetization-prepared rapid acquisition with gradient echo (3D MPRAGE) sequence (repetition time, TR: 3500 ms; echo time, TE: 3.5 ms; inversion time, TI: 1100 ms; field of view, FoV: 256 mm × 256 mm; 192 sagittal slices; flip angle, FA: 7 degrees; voxel size = 1 mm × 1 mm × 1 mm).

During the rsfMRI session, participants were instructed to stay awake, relax, and stare at the fixation point on the screen. In this session, T2-weighted images with blood oxygen level-dependent (BOLD) contrast were measured using a gradient echo-planar imaging (EPI) sequence (200 measurements; TR: 2500 ms; TE: 27 ms; FA: 77 degrees; FoV: 220 mm × 220 mm; matrix size = 64 × 64; voxel size = 3.4 mm × 3.4 mm × 3.4 mm).

### Pursuit Rotor Task

To examine visuomotor learning abilities before and after the cerebellar rTMS intervention, participants performed the pursuit rotor task, a widely used task to assess visuomotor coordination abilities (Harrington et al., 1990; Grafton et al., 1992; Knowlton et al., 2017; Lien et al., 2022).

During the task, participants were instructed to use the cursor to follow the target moving along the circular track with a drawing pen and tablet (WACOM Intuos; active area: 152 mm × 95 mm). If the cursor touches the target, the target color will turn light red as visual feedback. Performance at the following eight levels with different rotation speeds was assessed to determine the appropriate rotation speed level for visuomotor learning: 0.13, 0.16, 0.23, 0.3, 0.4, 0.5, 0.6, and 0.7 rotations per second (rps). During each session (pretest and posttest), participants moved from lower rotation speeds to higher rotation speeds, stopping at the level at which their maximum time on target was below 7.5 seconds (i.e., less than 50% successful tracking). The first level was used for practice and the other seven levels were used for testing. Six trials were performed at each testing level and each trial lasted 15 seconds. In each task session, both the total time until the cursor reached the target and the mean distance between the cursor and target center were recorded in each trial and are referred to as time on target and distance from target, respectively. These two measurements were used to generate behavioral performance indices (Lien et al., 2022).

### Repetitive Transcranial Magnetic Stimulation Interventions

Transcranial magnetic stimulation was performed using a Magstim Rapid^2^ stimulator (Magstim, United Kingdom) with an air-cooled figure-of-eight coil for active stimulation and an uncoated D70 alpha flat coil for sham intervention. Participants were kept blind to the coil by wearing an eye mask and hearing mechanical noise during the rTMS session. To determine the reference output intensity for the rTMS intervention, PT was assessed before the behavioral and rTMS sessions with computer software, namely, adaptive parameter estimation by sequential testing (Adaptive PEST for TMS)^1^. During the determination of PT, participants were instructed to open their eyes in a dark room and report the presence of phosphene in their visual fields after receiving active single-pulse TMS stimulation of their visual cortex (Marg and Rudiak, 1994). All participants underwent the rTMS intervention on the second day. In the cerebellar rTMS session, the rTMS interventions were administered in 1 Hz, 10 Hz and sham conditions, depending on the group (Langguth et al., 2008; Vasant et al., 2015) for 600 trials on the cerebellum. The location of stimulation was based on the midline cerebellum with an output intensity of 100% PT (Théoret et al., 2001; Argyropoulos and Muggleton, 2013). According to the 10-20 system, four markers, including Iz, Oz, O1, O2, were labeled and used to guide the stimulation location. The T1 images helped us determine more accurate site on scalp by estimating the distance between the target site and the markers. The approximately individual cerebellar target site located on 1-2 cm below the inion.

### Seed-Based Functional Connectivity Analysis

The rsfMRI data were analyzed using FSL v.5.0.11 (Functional MRI of the Brain Software Library), AFNI v.19.0.26 (Analysis of Functional NeuroImages), and SPM12 (Wellcome Department of Cognitive Neurology, London)^2^ with standard preprocessing methods. The standard preprocessing pipeline included the removal of the first ten volumes, slice timing correction, motion correction, boundary-based registration, spatial smoothing with a 6-mm full-width half-maximum (FWHM) intrinsic kernel, bandpass temporal filtering between 0.01 and 0.1 Hz, nuisance signal removal (white matter, cerebral spinal fluid, and head motion correction with the Friston 24-parameter model) and motion scrubbing with regression of framewise displacement (FD > 0.5 mm) spikes. Participants with head motion > 2 mm of translation or 2 ° of rotation were excluded.

To understand the relationship between baseline cerebro-cerebellar functional connectivity and visuomotor learning performance, we estimated the functional connectivity between six ROIs based on previous studies (Kelly and Strick, 2003; Morton and Bastian, 2006; Lu et al., 2007; Ishikawa et al., 2016). All the ROIs used in this study were shown in Supplementary Figure 2. The seed regions were located in the left primary motor cortex (L M1, MNI coordinates: - 41, - 20, 62; 6-mm radius sphere) and the cerebellum. We divided the cerebellum into five ROIs – right cerebellar cortex (R CbC, including lobules I-X, Crus I, and Crus II), left cerebellar cortex (L CbC), right deep cerebellar nucleus (R DCN, including dentate, fastigial, and interposed nucleus), left deep cerebellar nucleus (L DCN), and midline cerebellum (Mid Cb, including vermis VI-X, Vermis Crus I, and Vermis Crus II) – based on the SUIT atlas (Diedrichsen and Zotow, 2015). Furthermore, we explored the connections of the different DCNs for the efferent pathways. We divided the original seeds of the right and left DCN into six different subgroup seeds – right dentate nucleus (R DN), left dentate nucleus (L DN), right fastigial nucleus (R FN), left fastigial nucleus (L FN), left interposed nucleus (L IN), and right interposed nucleus (R IN) (Granziera et al., 2009; Diedrichsen et al., 2011; Kim et al., 2020). We used the same seed ROI in the primary motor cortex. Functional connectivity between the seven seed ROIs was estimated. All functional connectivity was calculated by the pairwise Pearson’s correlation coefficients between the BOLD time series extracted from each seed ROI and converted to z values using Fisher’s r-to-z transformation for the following comparisons.

### Behavior Data Analysis

All statistical analyzes were performed using the R packages “rstatix” (Kassambara, 2020), and “ppcor” (Kim, 2015).

To determine the accuracy of visuomotor learning, all behavioral data were transformed to logistic curves into represent visuomotor accuracy first. Then accumulation of the highest rotation speed while retaining high accuracy levels (more than 70%) was calculated to determine the appropriate rotation speed for visuomotor learning. After that, the estimated distance from the target was generated at this accuracy level as an indicator that represented the accuracy of the visuomotor performance. Furthermore, the mean amount of trial-to-trial variability (standard deviation of distance from the target across task sessions) was calculated as indicators representing the stability of visuomotor performance.

Behavioral improvement was estimated using the following formula:


(1) $$\mathrm{Improvement}=-\frac{\left(\mathrm{Posttest}-\mathrm{Pretest}\right)}{\mathrm{Pretest}}\times 100\%$$

Additionally, to determine the effect of rTMS on the learning performance of participants, we calculated the learning indices with the different individual indicators. One-way ANOVA with sham, 1 and 10 Hz groups were conducted to compare the learning performance in different groups and the effect sizes of the results were reported as generalized eta squared (η_2_) values. Partial Spearman’s rank correlation analysis with control covariate age was conducted for all the participants and groups to investigate the relationships between learning performance improvements and baseline functional connectivity following different rTMS interventions. The correlation coefficient was represented by rho. All statistical results were corrected by the correction for the false discovery rate (FDR) correction (alpha level was set < 0.05). Furthermore, power analyses were performed for all significant correlation results. We estimated the power (1-β) with assumption of α = 0.05, and two-tails hypothesis.

## Results

### Baseline Cerebro-Cerebellar Functional Connectivity and Visuomotor Learning

Neuroimaging data showed no significant differences in baseline functional connectivity between the different rTMS frequency groups by one-way ANOVA. Behavioral data showed that accuracy and stability performance in pretest among the three groups were not significantly different (see Supplementary Table 2). Furthermore, no significant correlation was found between the accuracy and stability indices (rho = −0.07, *p* = 0.676, conducted by Spearman’s rank correlation), suggesting that they contributed differently to visuomotor abilities. After cerebellar rTMS stimulation, the performance of visuomotor accuracy were significantly enhanced in the 10 Hz group and disrupted in the 1 Hz group compared to the sham group (F_2,35_ = 5.73, *p* = 0.007, η_2_ = 0.25). On the other hand, no significant group effect was found in the performance of visuomotor stability (F_2,35_ = 1.60, *p* = 0.216, η_2_ = 0.08). Our results suggested that cerebellar rTMS *via* frequency-dependent manner modulated visuomotor accuracy performance but not visuomotor stability performance. To determine the relation of baseline functional connectivity which with ROIs in the cerebellum and visuomotor learning, the partial correlation analyses were performed.

#### Significant Positive Correlation Between Baseline M1–Cerebellar Functional Connectivity and Improvements in Accuracy Performance

**FIGURE 1:**
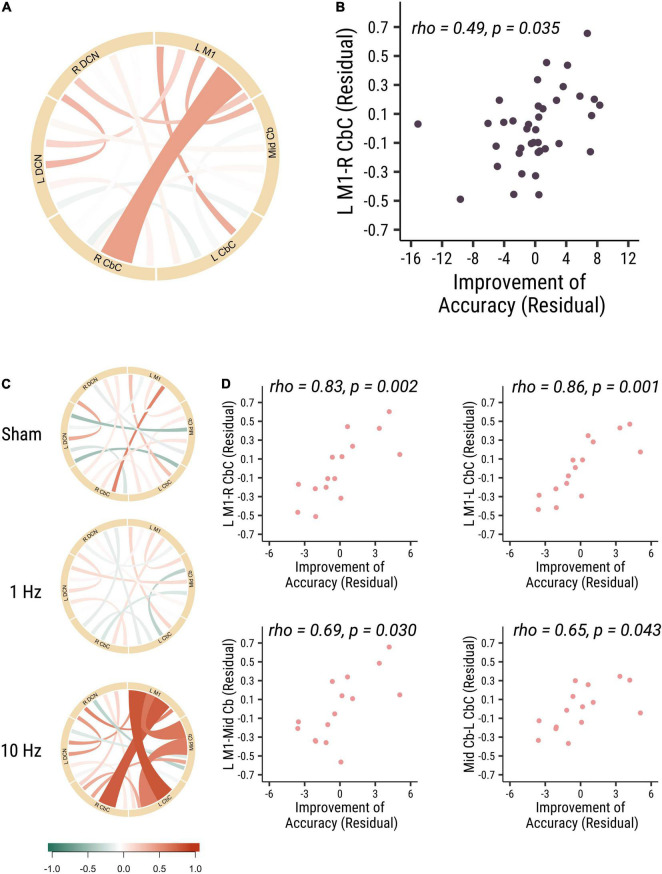
The baseline functional connectivity of M1-cerebellum positively correlated with the improvement in distance from target. **(A)** The correlation between different functional connectivity and the improvement in accuracy visuomotor performance in all participants. Link colors indicate partial Spearman’s rank correlation coefficient (rho) and link widths indicate the significance of correlation. The color bar shows the correlation coefficient scale. A thicker link shows a significant relationship (p-value < 0.05) between the functional connectivity of two ROIs and the accuracy improvements, and a thinner line shows no significant association between the functional connectivity and behavior. **(B)** The correlation between M1-R CbC functional connectivity and the accuracy improvements (rho = 0.49, *p* = 0.035). **(C)** The correlation between different functional connectivity and the accuracy improvements among three different cerebellar rTMS groups. **(D)** The correlation between functional connectivity between L M1-R CbC (rho = 0.83, *p* = 0.002; top right panel), L M1-L CbC (rho = 0.86, *p* = 0.001; top left panel), L M1-Mid Cb (rho = 0.69, *p* = 0.03; bottom right panel) and Mid Cb-L CbC (rho = 0.65, *p* = 0.043; bottom left panel) functional connectivity and the accuracy improvements in the 10 Hz rTMS group. Each point is one participant. Each point is one participant. (L M1 = left motor cortex; R CbC = right cerebellar cortex; L CbC = left cerebellar cortex; R DCN = right deep cerebellar nucleus; L DCN = left deep cerebellar nucleus; Mid Cb = midline cerebellum).

The results did not show significant correlations between all measures of baseline functional connectivity and baseline visuomotor performance. And then we analyzed the correlation of baseline functional connectivity and the change of the visuomotor accuracy performance. Functional connectivity of left M1 to right cerebellar cortex (L M1-R CbC) was positively correlated with accuracy improvements in all participants (rho = 0.49, p = 0.035, Cohen’s *d* = 0.887; see Figures 1A,B). Specifically, in the 10 Hz rTMS group, the functional connectivity between three ROIs (L M1-R CbC, L M1-L CbC, and L M1-Mid Cb), which are among the cerebro-cerebellar pathways, was found to be positively correlated with accuracy improvements (L M1-L CbC: rho = 0.86, *p* = 0.001, Cohen’s *d* = 0.996; L M1-R CbC: rho = 0.83, *p* = 0.002, Cohen’s *d* = 0.988; L M1-Mid Cb: rho = 0.69, *p* = 0.03, Cohen’s *d* = 0.859). Functional connectivity between ROIs within the cerebellum (Mid Cb-L CbC) was also positively correlated with accuracy improvements (rho = 0.65, *p* = 0.043, Cohen’s *d* = 0.792) (see Figures 1C,D). In summary, these results suggested that participants who had higher baseline functional connectivity of L M1-R CbC, L M1-L CbC, L M1-Mid Cb and Mid Cb-L CbC would have larger performance improvement in visuomotor accuracy. That is, the higher baseline functional connectivity in the afferent cerebro-cerebellar pathways may highly associated with higher accuracy improvements.

#### Significant Negative Correlation Between the Baseline Deep Cerebellar Nuclei—M1 Functional Connectivity and Improvement in Stability Performance

**FIGURE 2:**
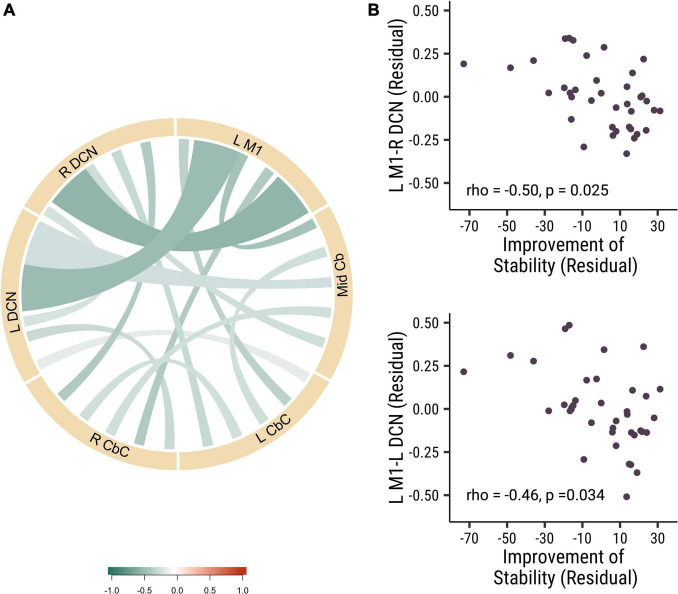
The baseline functional connectivity of M1 to deep cerebellar nuclei was corelated with the improvement in trial-to-trial variability. **(A)** The correlation of different functional connectivity and the stability improvement in all participants. **(B)** The correlation between the functional connectivity of L M1-R DCN (rho = –0.50, *p* = 0.025; top panel) and L M1- L DCN (rho = –0.46, *p* = 0.034; bottom panel) functional connectivity and the stability improvements. Each point is one participant. (L M1 = left motor cortex; R CbC = right cerebellar cortex; L CbC = left cerebellar cortex; R DCN = right deep cerebellar nucleus; L DCN = left deep cerebellar nucleus; Mid Cb = midline cerebellum).

In our study, we did not find any significant correlation between the baseline visuomotor performance stability and the strength of functional connectivity at baseline. Then we analyzed the correlation of baseline functional connectivity and the change of the visuomotor stability performance. Interestingly, we found that the baseline functional connectivity of L M1-L DCN (rho = −0.46, *p* = 0.034, Cohen’s *d* = 0.839) and L M1-R DCN (rho = −0.50, *p* = 0.025, Cohen’s *d* = 0.907) was negatively correlated with stability improvements in all participants (see Figure 2). There were no significant correlations between functional connectivity and stability improvements between the different rTMS frequency groups. These results suggested that participants who had lower baseline cortical-DCN functional connectivity would show greater improvements in stability in the visuomotor task.

### Baseline Functional Connectivity From the Deep Cerebellar Nucleus to the Motor Cortex and Visuomotor Learning

**FIGURE 3:**
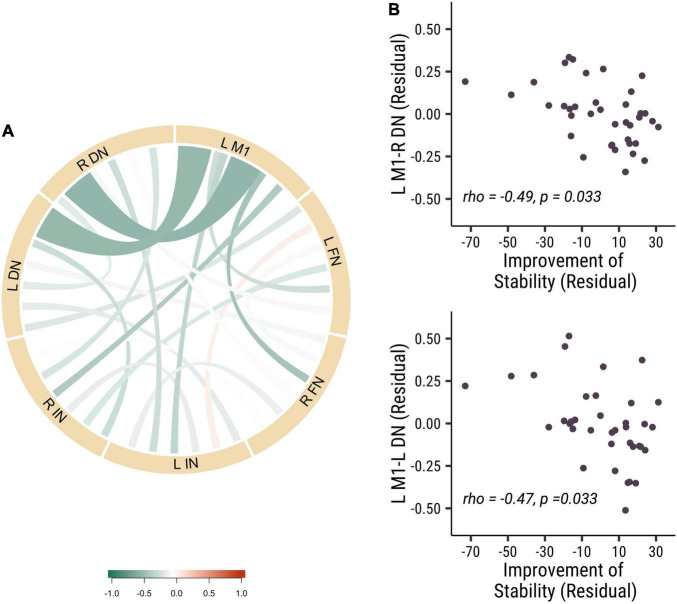
The baseline functional connectivity of M1 to dentate, fastigial, and interposed nucleus, corelated with the improvement of stability. **(A)** The correlation of different functional connectivity and the stability improvements in all participants. **(B)** The correlation between functional connectivity of L M1-R DN (rho = –0.49, *p* = 0.033; top panel) and L M1-L DN (rho = –0.47, *p* = 0.033; bottom panel) functional connectivity and the improvement of stability. Each point is one participant. (L M1 = left motor cortex; R DN = right dentate nucleus; L DN = left dentate nucleus; R FN = right fastigial nucleus; L FN = left fastigial nucleus; R IN = right interposed nucleus; L IN = left interposed nucleus).

To further investigate the role of the different nuclei among DCN in the stability of visuomotor performance, we divided the DCN into three seeds: dentate nucleus (DN), fastigial nucleus (FN) and interposed nucleus (IN). We measured the strength of baseline functional connectivity between these different seeds and the left primary motor cortex. We investigated the relation between these baseline functional connectivity and stability improvements in visuomotor performance. Results revealed that the functional connectivity of L M1-R DN (rho = −0.49, *p* = 0.033, Cohen’s *d* = 0.899) and L M1-L DN (rho = −0.47, *p* = 0.033, Cohen’s *d* = 0.868) was negatively correlated with improvements in stability (see Figure 3). These results indicated that the dentate nucleus might be more critical in regulating the stability of visuomotor performance than the fastigial and interposed nuclei. Those results demonstrated that lower baseline functional connectivity in dentato-cortical pathways was correlated with improvements in stability indices. The amount of stability improvements is highly related to the functional connectivity of the efferent dentato-cerebral circuits but not the afferent cerebro-cerebellar circuits.

In summary, those results showed that the effects of baseline functional connectivity in the afferent and efferent pathways were correlated in an opposite manner with visuomotor learning, which may suggest that there are different roles of the cerebellum in visuomotor learning with distinctive baseline connectivity of cerebellar pathways.

## Discussion

**FIGURE 4:**
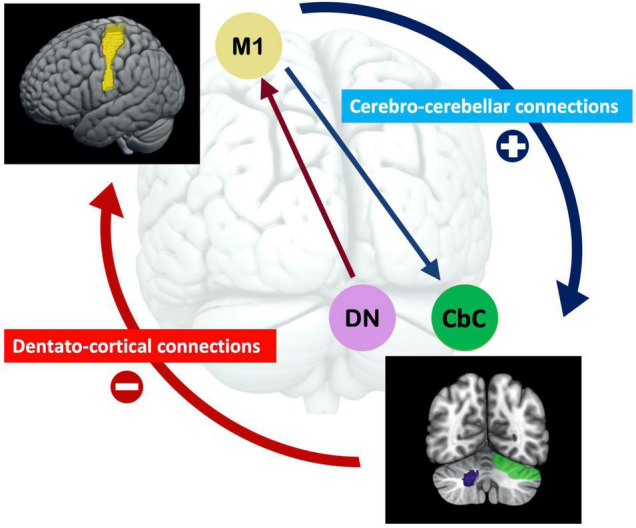
The opposite effect of the baseline afferent and efferent pathways of functional connectivity of the cerebellum for visuomotor learning. The higher baseline functional connectivity of the cerebro-cerebellar pathway, the better accuracy learning performance; and the lower baseline functional connectivity of the cerebellar-cerebral pathway, the better stability learning performance in the visuomotor task. Functional connectivity in the efferent dentato-cerebral pathway is related to the dentato-thalamo-cortical pathway, in which the dentate nucleus reduces inhibition signals from Purkinje cells. The disinhibition of these inhibitory projections is essential for visuomotor learning. (M1 = motor cortex; DN = dentate nucleus; CbC = cerebellar cortex).

The baseline rsfMRI results showed the differential contributions of the baseline corticocerebellar functional connectivity that corresponded to the afferent and efferent circuits to visuomotor learning abilities. Higher baseline functional connectivity of L M1-R CbC in afferent cerebro-cerebellar pathways was positively correlated with improvements of accuracy in visuomotor learning. The baseline functional connectivity of L M1-R DCN and L M1-L DCN in efferent cerebellar-cerebral pathways was negatively correlated with improvements of stability in visuomotor learning. Furthermore, we demonstrated that the dentate nucleus plays an important role in cerebellar to cerebral efferent pathways and was correlated with the stability of visuomotor performance, which is consistent with many studies examining the dentate nucleus (Habas, 2010; Kuper et al., 2014). Our results suggested that the higher the baseline functional connectivity in the afferent cerebro-cerebellar pathway, the better the accuracy of learning performance, and the lower the baseline functional connectivity of the efferent cerebellar-cerebral pathway, the better the stability of learning performance in the visuomotor task. Interestingly, opposite results were observed for baseline functional connectivity in the afferent and efferent pathways of the cerebellum and their correlations with visuomotor learning (see Figure 4). Opposite dynamic changes in the cerebellar cortex and dentate nucleus support our results and indicate that their neuronal mechanisms are also different (Floyer-Lea and Matthews, 2004).

In addition, our results reveal that the cerebellar afferent and efferent pathways may contribute visuomotor learning differentially. In visuomotor coordination, the involved cerebellar pathways are the cerebro-ponto-cerebellar connections as afferent cerebro-cerebellar pathways and the dentato-thalamo-cortical connections as efferent cerebro-cerebellar pathways. Cerebro-ponto-cerebellar connections carry motor command signals from the motor cortex and project to the cerebellar cortex, and those Purkinje cells project to the dentate nucleus where sensory prediction errors are generated (Streng et al., 2018; Welniarz et al., 2021). Dentato-thalamo-cortical connections carry sensory prediction error signals back to the motor cortex and modulate new motor commands for visuomotor coordination (Kawato et al., 1987; Honda et al., 2018). Those studies suggest that the functional connectivity in the afferent cerebro-cerebellar pathway, which carries the motor command from the motor cortex to the cerebellum, may mainly relate to the signal inputs of visuomotor learning. The functional connectivity in the efferent cerebro-cerebellar (dentato-thalamo-cortical) pathway is indicative of a reduction in Purkinje cell inhibition of DCN neurons (Ishikawa et al., 2014), which may provide error signals back to motor cortex for visuomotor learning. It suggests that the afferent and efferent pathways in the cerebellum are anatomically and functionally distinct. Therefore, it is important to involve these two functional networks be analyzed separately in the context of visuomotor learning.

### Increased Baseline Functional Connectivity in Afferent Cerebro-Cerebellar Pathways Related to Learning Acquisition

In our study, we showed that individuals who had higher baseline cerebro-cerebellar functional connectivity (L M1-R CbC) had better visuomotor coordination among all participants. Our participants were all right-handed, which means that the left motor cortex dominated and the cerebro-cerebellar projection crossed over to the right cerebellar cortex. The cerebro-cerebellar pathway carries the motor command from the motor cortex to the cerebellum for visuomotor learning acquisition (Kawato et al., 1987; Honda et al., 2018). In our subgroup analysis, participants who received excitatory 10 Hz cerebellar rTMS with higher functional connectivity of the cerebrocerebellar (L M1-R CbC, L M1-L CbC, L M1-Mid Cb) and intracerebellar (Mid Cb-L CbC) functional connectivity at baseline had a better improvement in visuomotor performance. Compared to the L M1-R CbC connectivity in all participants, the 10 Hz cerebellar rTMS group was not restricted to the right side of the cerebellum, as the left and midline cerebellar and even intracerebellar (Mid Cb-L CbC) connectivity were significantly changed with visuomotor performance. These results suggested that the strength of functional connectivity of not only cerebro-cerebellar but also intracerebellar connections was correlated with visuomotor learning. Several studies have shown that increased cerebro-cerebellar functional connectivity became positively correlated after visuomotor coordination tasks (Miall et al., 2001; Muller et al., 2002; Della-Maggiore et al., 2017), especially in the early stages of visuomotor learning (Muller et al., 2002; Floyer-Lea and Matthews, 2004). Although we did not calculate the change in functional connectivity, we demonstrated that increased baseline functional connectivity was related to better visuomotor performance and was even a predictor of improvements in visuomotor performance.

### Decreased Baseline Functional Connectivity in the Efferent Dentato-Cortical Functional Pathway Related to the Inhibitory Signal of the Dentate Nucleus

Our results showed that the functional connectivity of L M1-R DN and L M1-L DN was negatively correlated with improvements in stability. We demonstrated that a decrease in baseline functional connectivity in the efferent dentato-cortical pathway is related to improvements in the stability of visuomotor learning. The dentate nucleus is involved in planning, executing voluntary movements, sensory processing, and high cognitive function (Guell et al., 2020). Most efferent fibers synapse in the dentate nuclei before reaching extracerebellar structures such as the cerebral cortex (Haines and Dietrichs, 2012). The dentate nucleus sends inhibitory GABAergic fibers through the superior cerebellar peduncle to the contralateral red nucleus (Ishikawa et al., 2014). Initial burst activity in dentate nucleus cells is generated by reduced inhibitory signals from Purkinje cells (Ishikawa et al., 2014). In other words, the disinhibition mechanism in the dentate nucleus can play a key role in motor learning by suppressing the intensive inhibitory drive from Purkinje cells (Ishikawa et al., 2014). Therefore, we suggest that efferent inhibitory GABAergic fibers may explain these findings. Our results may provide more evidence to support the disinhibition mechanism of the dentate nucleus and associated with the reduction of dentato-thalamo-cortical functional connectivity. Disinhibition of these inhibitory projections regulates the stability of performance in visuomotor coordination.

Moreover, we demonstrated that the dentate nucleus, and not the fastigial or interposed nuclei, is critical for regulating the stability of visuomotor performance. The dentato-thalamo-cortical pathway carries sensory prediction errors from the cerebellum to the cortex and serves as a forward internal model that compares motor commands and sensory feedback to generate sensory prediction errors for visuomotor learning (Ishikawa et al., 2016; Streng et al., 2018; Tzvi et al., 2021; Welniarz et al., 2021). A recent study demonstrated that dentate nuclei cell firing rates could predict future inputs to the cerebellum (Tanaka et al., 2019). Recent functional gradient imaging studies have shown that the dentate nucleus influences multiple functional territories, including default mode, salience-motor, and visual brain networks (Habas, 2010; Guell et al., 2018, 2020; Lin et al., 2020), which means that the dentate nucleus represents a wide range of macroscale functional categories in the brain. The dentate nucleus and cerebellar cortex become activated during visuomotor adaptation tasks in fMRI studies (Habas, 2010; Kuper et al., 2014). A neuromodulation study with stroke patients showed that cerebellar transcranial direct current stimulation of the dentate nuclei can improve post-stroke balance rehabilitation (Rezaee et al., 2020). We highlighted the role of the dentate nucleus and the efferent dentato-thalamo-cortical pathways in modulation of visuomotor function.

This study had some limitations. First, rsfMRI was performed before the visuomotor task and we did not calculate the change in functional connectivity. Because resting-state activity supports introspective thought, processes previous experiences, and supports responses to future events (Miall and Robertson, 2006; Raichle and Snyder, 2007), motor learning can modulate subsequent activity within resting networks (Logothetis et al., 2001; Albert et al., 2009). While our study demonstrated that baseline functional connectivity is related to visuomotor performance and may predict visuomotor learning abilities (Mawase et al., 2017), follow-up neural imaging studies involving pre- and post-visuomotor learning observations are essential to investigate the role of afferent and efferent cerebro-cerebellar pathways in visuomotor learning. Second, we choose several brain areas for research interesting for the visuomotor coordination pathways, such as M1 and cerebellum. Indeed, there are many different brain regions and connections changes in functional connectivity, which beyond our aims of study. Third, we assume that inhibitory GABAergic dentato-thalamo-cortical pathways are the main reason for the negative relationship between decreased functional connectivity and visuomotor learning performance. Future studies, including different neuroimaging observations, such as cerebellar magnetic resonance spectroscopy, will be helpful in understanding the neurochemical mechanisms underlying cerebellar visuomotor learning.

In conclusion, our study demonstrated that the cerebellar cortex and dentate nucleus and its afferent and efferent connectivity work differently during visuomotor coordination. The increased baseline functional connectivity in the afferent cerebro-cerebellar pathways is related to greater improvements in accuracy. Baseline functional connectivity in the efferent dentato-thalamo-cortical pathways carries inhibitory signals and regulates visuomotor coordination. Therefore, decreased baseline functional connectivity in the efferent dentato-thalamo-cortical pathways is related to improved stability in visuomotor performance. We emphasize that the dentate nucleus and its dentato-cortical functional connectivity may have a crucial effect on visuomotor coordination. We can predict visuomotor learning abilities by testing baseline cerebro-cerebellar functional connectivity (Mawase et al., 2017). The functional dissociation of the cerebellar cortex and dentate nucleus and their connections indicate distinct mechanisms in the cerebellum with respect to visuomotor learning. Furthermore, the cerebrocerebellar circuit neuromodulation, such as cerebellar rTMS or tDCS, is effective for the control of motor symptoms in patients with cerebellar lesions or visuomotor deficits (Miterko et al., 2019). Our study provides evidence for using rTMS as an intervention technique in populations with visuomotor deficits or cerebellar disorders.

## Data Availability Statement

The raw data supporting the conclusions of this article will be made available by the authors, without undue reservation.

## Ethics Statement

The studies involving human participants were reviewed and approved by Institutional review board of National Yang Ming Chiao Tung University. The patients/participants provided their written informed consent to participate in this study.

## Author Contributions

Y-CL, YL, S-HL, C-PL and L-HC designed the experiments. YL, S-HL, and L-HC performed the experiments and analyzed the data. Y-CL, YL, S-HL, Y-CK, C-CH, C-PL and L-HC wrote the manuscript. All authors contributed to the article and approved the submitted version.

## Conflict of Interest

The authors declare that the research was conducted in the absence of any commercial or financial relationships that could be construed as a potential conflict of interest.

## Publisher’s Note

All claims expressed in this article are solely those of the authors and do not necessarily represent those of their affiliated organizations, or those of the publisher, the editors and the reviewers. Any product that may be evaluated in this article, or claim that may be made by its manufacturer, is not guaranteed or endorsed by the publisher.

## Footnotes

1
https://www.clinicalresearcher.org

2
http://www.fil.ion.ucl.ac.uk/spm
