# Supplementary material for: Baseline Cerebro-Cerebellar Functional Connectivity in Afferent and Efferent Pathways Reveal Dissociable Improvements in Visuomotor Learning
Source: Front Neurosci. 2022 May 26;16:904564. doi: 10.3389/fnins.2022.904564 (PMC9204583; doi:10.3389/fnins.2022.904564)
Supplement: Supplementary file 1 [file Table_1.DOCX]

Supplementary Material

## Supplementary Tables

|  | **Sham** | **1 Hz** | **10 Hz** |
| --- | --- | --- | --- |
| ***N*** | 11 | 12 | 15 |
| **Gender** | 5 female, 6 male | 7 female, 5 male | 9 female, 6 male |
| **Average age** | 23.4 | 22.2 | 22.8 |
| **Average education years** | 16.8 | 16.2 | 16.7 |

**Supplemental Table 1. Subject Table.**

| **Accuracy Performance (Baseline)** | | |
| --- | --- | --- |
| rTMS Group | mean (pixel) ± standard error | *p*-value |
| Sham | 19.75 ± 0.13 | *F_2,35_ =* 0.98*, p =* 0.387, η­_2_ = 0.05 |
| 1 Hz | 20.01 ± 0.15 |  |
| 10 Hz | 19.93 ± 0.10 |  |
| **Accuracy Performance (Improvement)** | | |
| rTMS Group | mean (%) ± standard error | *p*-value |
| Sham | 13.69 ± 1.24 | *F_2,35_ =* 5.73*, p =* 0.007**, η­_2_ = 0.25 |
| 1 Hz | 9.03 ± 1.60 |  |
| 10 Hz | 14.30 ± 0.80 |  |
| **Stability Performance (Baseline)** | | |
| rTMS Group | mean (pixel) ± standard error | *p*-value |
| Sham | 2.62 ± 0.25 | *F_2, 35_ =* 0.60*, p =* 0.557, η­_2_ = 0.03 |
| 1 Hz | 2.66 ± 0.28 |  |
| 10 Hz | 2.36 ± 0.15 |  |
| **Stability Performance (Improvement)** | | |
| rTMS Group | mean (%) ± standard error | *p*-value |
| Sham | 6.01 ± 7.85 | *F_2, 35_=* 1.60*, p =* 0.216, η­_2_ = 0.08 |
| 1 Hz | 21.69 ± 5.13 |  |
| 10 Hz | 18.89 ± 5.93 |  |

**Supplemental Table 2. Behavioral performance in each group.** The table showed the mean and standard error of both accuracy and stability performance in pretest, and the mean and standard error of both accuracy and stability improvement. The one-way ANOVA result showed that a significant main effect of rTMS group was found in accuracy improvement. The effect sizes were represented by generalized eta squared (η­_2_).

**Supplementary Figures**

##
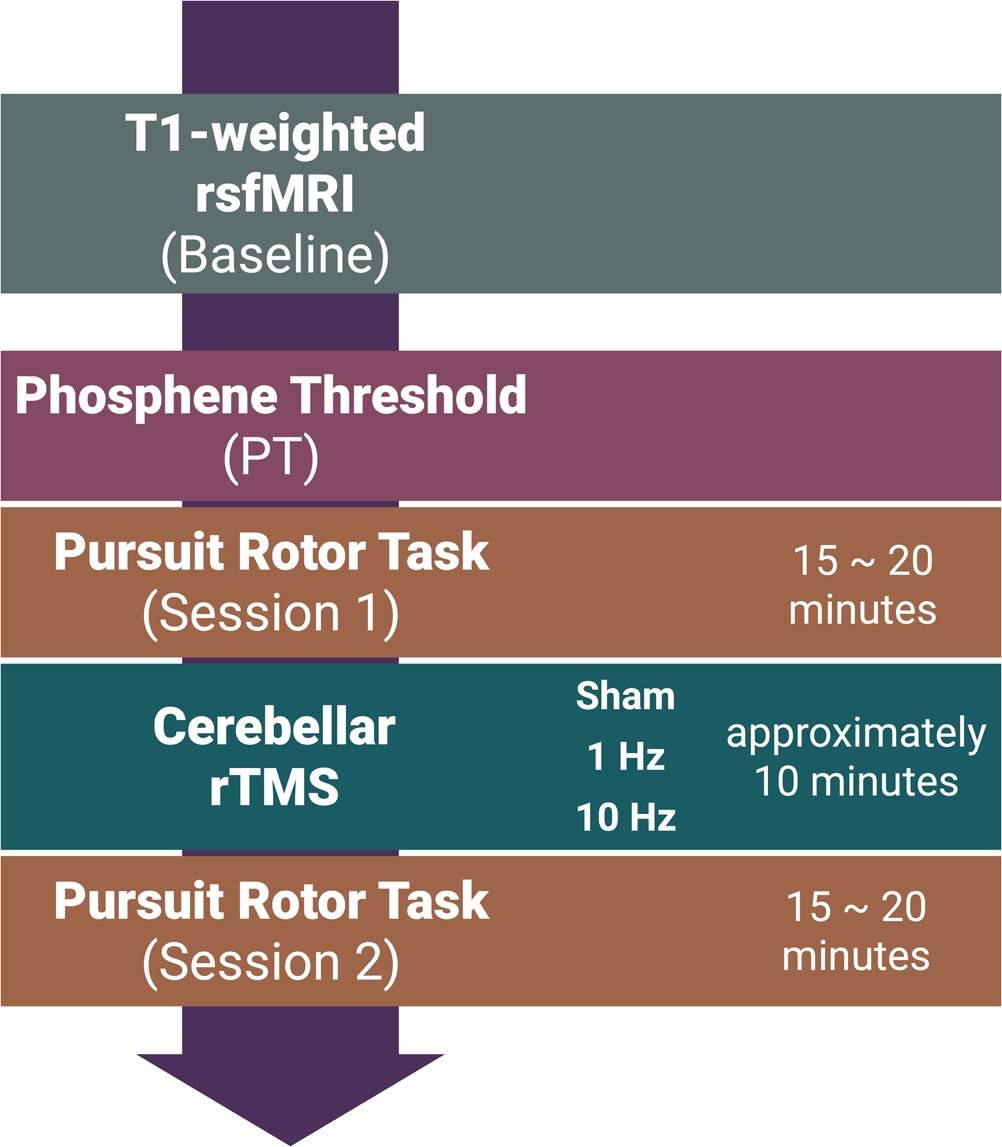


**Supplementary Figure 1. Experimental Procedure.** Structural imaging and resting state fMRI were performed to determine the stimulate target region and to measure the functional connectivity. We also measured the phosphene threshold (PT) at baseline to have the reference output intensity of rTMS intervention. On the second day, the participants were randomly assigned to one of the three groups: 1 Hz, 10 Hz, or sham group to receive different frequencies cerebellar rTMS interventions. All the interventions contained total 600 pulses and whole the rTMS session was completed within approximately 10 minutes. Performance on the behavioral task was assessed before and after the cerebellar rTMS intervention immediately.


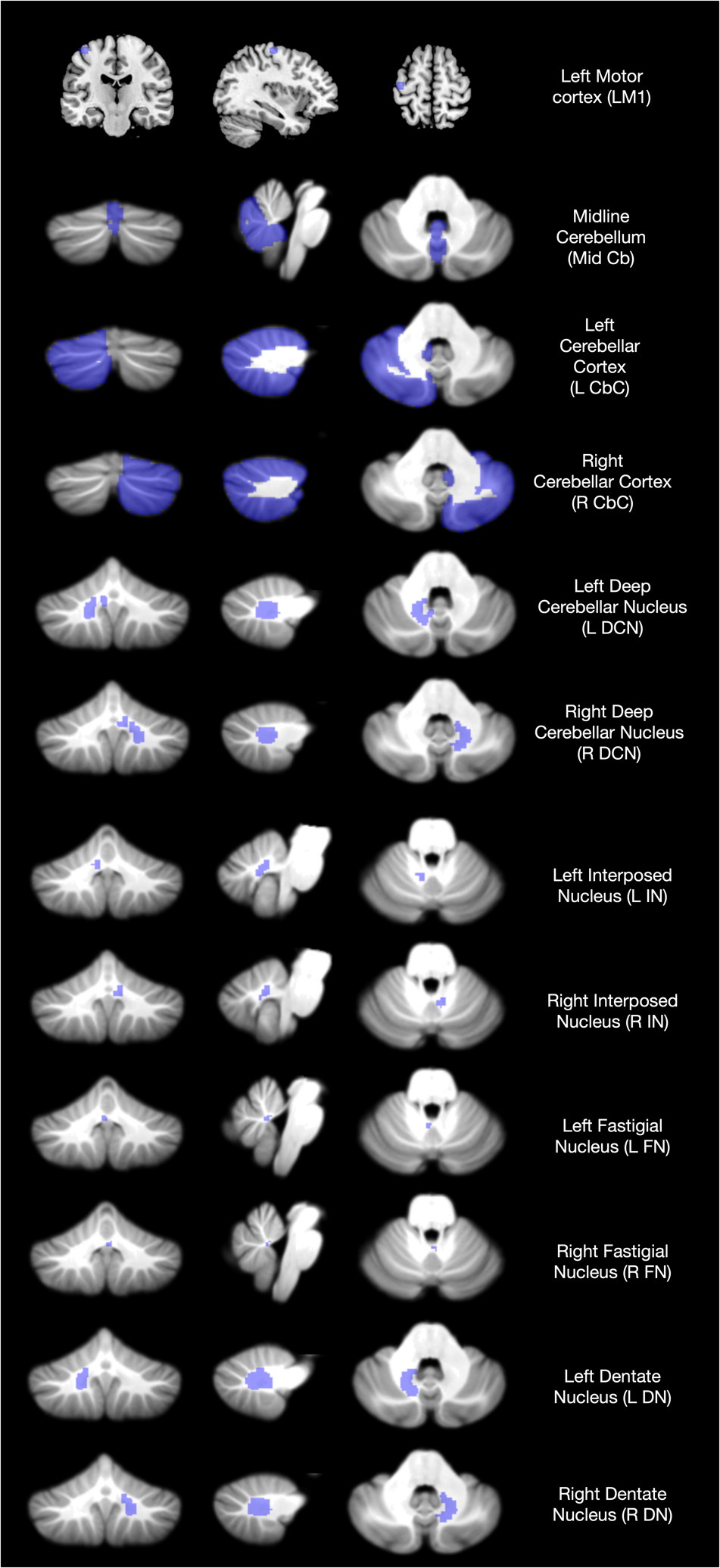


**Supplementary Figure 2. ROIs for seed-based analysis.** Structural imaging and anatomical label (blue) for ROIs which were used for seed-based analysis in this study.
